# Contrasting patterns of prehistoric human diet and subsistence in northernmost Europe

**DOI:** 10.1038/s41598-018-19409-8

**Published:** 2018-01-18

**Authors:** Mirva Pääkkönen, Auli Bläuer, Bjørnar Olsen, Richard P. Evershed, Henrik Asplund

**Affiliations:** 1 Department of Archaeology, University of Turku, Turku, FI-20014 Finland; 2 Green Technology, Natural Resources Institute Finland, Turku, FI-20520 Finland; 3 Department of Archaeology, History, Religious Studies and Theology, UiT - The Arctic University of Norway, Tromsø, N-9037 Norway; 4 Organic Geochemistry Unit, School of Chemistry, University of Bristol, Bristol, BS8 1TS United Kingdom

## Abstract

Current archaeological evidence indicates the transition from hunting-fishing-gathering to agriculture in Northern Europe was a gradual process. This transition was especially complex in the prehistoric North Fennoscandian landscape where the high latitude posed a challenge to both domestic animal breeding and cereal cultivation. The conditions varied, the coastal dwellers had access to rich marine resources and enjoyed a milder climate due to the Gulf Stream, while those living in the inland Boreal forest zone faced longer and colder winters and less diversity in animal and plant resources. Thus, the coastal area provided more favourable conditions for early agriculture compared to those found inland. Interestingly, a cultural differentiation between these areas is archaeologically visible from the late 2^nd^ millennium BC onwards. This is most clearly seen in regionally distinct pottery styles, offering unique opportunities to probe diet and subsistence through the organic residues preserved in ceramic vessels. Herein, we integrate the lipid biomarker, compound-specific stable carbon isotopes (δ^13^C), and zooarchaeological evidence to reveal culturally distinct human diets and subsistence patterns. In northern Norway, some of the coastal people adopted dairying as part of their subsistence strategy, while the inhabitants of the interior, in common with northern Finland, continued their hunter-gatherer-fisher lifestyles.

## Introduction

**Figure 1:**
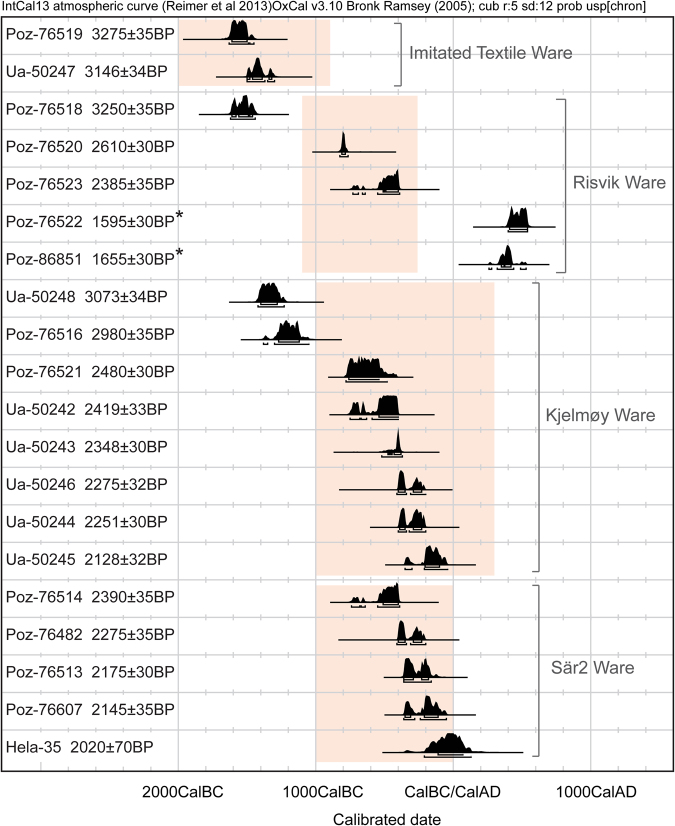
AMS dates from the crusts on the vessels. The AMS dates from crusts of the studied vessels show that all of the vessels dated to the expected periods, with the exception of one of the Risvik vessels (SÄR73), which was younger than expected, dating to 400–540 cal AD (with a 95.4% probability). Because of this exceptional date, the sample was re-dated as a duplicate, but the result did not change considerably (260–280 cal AD (1.9%), 320–440 cal AD (87.8%), and 490–530 cal AD (5.7%)). All dates are from this study except for Hela-35^10^. The calibration used the OxCal v3.10 program^46,47^ and the IntCal13 calibration data^48^. * = Two different datings for vessel SÄR73. Rectangles indicate typical dating range for each ceramic type.

Summer days are long in Fennoscandia (Norway, Sweden, Finland, and Kola Peninsula), but north of the Arctic Circle the sun does not rise above the horizon during the winter months. Still, North Fennoscandia was populated soon after the last ice sheet retreated from the area around 10 000–9000 BC. During most of the Stone Age (10 000–1800 BC) a mobile hunter-gatherer lifestyle was dominated here^1,2^. However, by the end of this era, more sedentary settlement patterns developed in some coastal areas of the north^3^. During the Early Metal Period (1800–1 BC) the Fennoscandian cultural landscape differentiated. Communities along the western coast of North Norway and in the Gulf of Bothnia adopted elements of an agricultural way of life and increasingly oriented themselves toward South Scandinavian societies. The communities in the interior and north-eastern Fennoscandia continued their hunting and gathering lifestyle and became more involved with eastern and central Russian exchange network. This differentiation is reflected in the pottery styles from this period^3,4^. The tradition of using asbestos-tempered ceramics is widespread in North Fennoscandia and became especially common in the region around 2000 BC. At the end of the 2^nd^ millennium BC, the asbestos pottery used in the area differentiated into two distinct styles, termed Risvik (1100–270 BC, main period 800–400 BC) and Kjelmøy pottery (1000 BC–300 AD, main period 900–100 BC, Fig. 1)^3^. Sites with Risvik pottery are located on the northwest parts of the Norwegian coast, whereas the Kjelmøy pottery sites are mainly distributed in the interior and northernmost coastal area of North Fennoscandia^5,6^.

Using only the sparse archaeological record, Risvik pottery appears connected with communities that integrated agriculture into their economies and exhibited increased cultural affiliations with the Nordic Bronze Age societies further south in Scandinavia. In contrast, Kjelmøy pottery is associated with communities who maintained a hunter-gatherer way of life^3^. In addition to Kjelmøy and Risvik, other Early Metal Period pottery types are found in North Fennoscandia. This includes ceramics that exhibit stylistic and chronological affinities with Kjelmøy pottery, such as mica and shell tempered pottery found in north-eastern Norway and the more heterogeneous Säräisniemi 2 (Sär 2, 1000–1 BC) pottery used by the hunter-gatherers in North Finland^4^. Also for the latter group, other means of tempering than asbestos have been identified. Imitated Textile (IT, 2000–900 BC) pottery represents an earlier tradition of asbestos ceramics (Fig. 1). The latter occurs widely in most parts of North Fennoscandia and has been connected with a previous more uniform hunter-gatherer lifestyle^7–10^.

The subsistence patterns of groups living in the North Fennoscandia during the Early Metal Period can be explored through studying ceramics vessels, and more precisely, as done here, by studying organic residues found from Arctic Norway and North Finland. Previous studies have shown that lipids survive widely absorbed in the ceramic matrix of archaeological vessels from South Fennoscandia with a range of biomarkers offering unique opportunities to probe spatial and temporal changes in vessel use and, by extension, examine the different food procurement strategies. In particular, preserved fatty acids have been used to determine the use of pottery for the processing of animal products, while δ^13^C values of palmitic acid (C_16:0_) and stearic acid (C_18:0_) homologues are used to identify the source of fats, specifically, ruminant adipose, non-ruminant adipose, and dairy fats^11^. Further, specific biomarkers originating from aquatic organisms can be used to detect lipid residues of marine and freshwater organisms, notably: ω-(*o*-alkylphenyl)alkanoic acids (APAAs), isoprenoid fatty acids (IFAs), phytanic acid, pristanic acid, 4,8,12-trimethyltetradecanoic acid (4,8,12-TMTD), and dihydroxy fatty acids (DHYAs)^12–18^.

The current model suggests that while all the societies of the Early Metal period produced pottery, those along the climatically milder western coast of North Norway became orientated more toward agriculture and adopted dairying and cereal cultivation as part of their subsistence. At the same time, the communities of interior and north-eastern areas continued their hunter-gatherer-fisher way of life^3^. Herein, we test the aforementioned culturally-specific model for diet and subsistence through the lens of absorbed organic residues found in pottery. The distinct and differing pottery styles offer unique opportunities to probe subsistence patterns through examination of the organic residues preserved in the vessels. Building on previous research based on archaeological and zooarchaeological records, we seek to develop new understandings of food procurement strategies in Europe’s far north during a time of major economic change. Thus, in addition to studying organic residues, we compare these findings with the results obtained from zooarchaeological analyses in order to have better understanding of subsistence patterns of coastal early agrarian communities, coastal hunter-gatherers, and interior hunter-gatherers. To achieve this goal, we assembled a substantial collection of pottery comprising Risvik (putative agrarian), Kjelmøy, mica and shell tempered ware, IT, and Sär 2 (hunter-gatherer) pottery types derived from both inland and coastal settlements.

## Results

**Figure 2:**
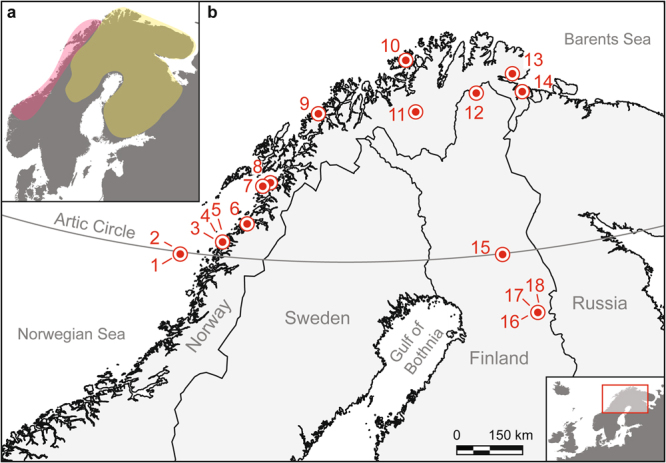
Panels showing the geographical distribution of the asbestos tempered pottery and the studied sites. (**a**) Red indicates the distribution of agrarian Risvik pottery and yellow the distribution of Sär 2 and asbestos tempered hunter-gatherer pottery in Fennoscandia. (**b**) Map of the study area showing the locations of the target sites in Arctic Norway and North Finland. 1 = Trӕna Kirkhellaren (R), 2 = Trӕna Røsnesvalen (R), 3 = Meløy Nedre Valla (R), 4 = Meløy Solheim Mesøy (R), 5 = Meløy Texmoen (R), 6 = Bodø Skålbunes (R), 7 = Steigen Bo (R), 8 = Hamarøy Uteid (R), 9 = Tromsø Sandvika (R), 10 = Sørøysund Sandbukt Sørøy (IT), 11 = Kautokeino Virdejávri 106 (K), 12 = Utsjoki Guatniljärvi (S2), 13 = Nesseby Mortensnes (K), 14 = Sørvaranger Mestersanden (K, Msh), 15 = Kemijärvi Neitilä 4 (S2), 16 = Suomussalmi Kalmosärkkä N (S2), 17 = Suomussalmi Kalmosärkkä S (S2), 18 = Suomussalmi Kellolaisten Tuli (S2), where IT = Imitated Textile Ware, K = Kjelmøy pottery, Msh = mica and shell tempered ware, S2 = Sär2 Ware, and R = Risvik pottery. The sherd from Tromsø Sandvika only resembled Risvik pottery. Map created using MapInfo Professional 12.0.3 (www.pitneybowes.com) and Adobe Illustrator CS6 (www.adobe.com).

Fifty eight potsherds from archaeological sites in North Finland and Arctic Norway (Fig. 2) were selected for analysis. All the studied sites were located near water bodies, either at the ocean coast or along interior lakes and rivers. The studied vessels were all low temperature-fired wares tempered by either asbestos, mica, talc, soapstone, or shell. Lipid extracts were analysed using gas chromatography (GC). The lipid extracts of 46 vessels were studied further using gas chromatography-mass spectrometry (GC-MS), and GC-combustion-isotope ratio MS (GC-C-IRMS), including those from: 10 Risvik pottery sherds and a wide range of sherds from hunter-gatherer sites, namely, 15 from Kjelmøy sherds, 6 sherds tempered with mica and shell, 3 sherds from IT pottery, and 12 Sär 2 pottery sherds (Table 1, Supplementary Table S1).

**Table 1:** Summary of organic residue analysis of studied pottery based on lipid biomarkers and δ^13^C values. The values represent number of vessels with identified fatty acid content. ? = uncertain identification. More detailed information is presented in Supplementary Table S1.

**Pottery type**	**Coastal agcricultural groups**	**Coastal hunter-gatherer groups**			**Interior hunter-gatherer groups**	
	*Risvik Pottery*	*Kjelmøy Pottery*	*Mica and shell tempered Ware*	*Imitated Textile Ware*	*Kjelmøy Pottery*	*Sär 2 Ware from inland Finland*
Aquatic fats	2	5	5	2		
Aquatic? fats	2					
Aquatic fats, Ruminant carcass fats?				1		1
Aquatic fats?, Other terrestrial fats?		2				2
Dairy fats	3					
Dairy fats, Aquatic fats?	3					
Ruminant carcass fats		1			5	4
Other terrestrial fats?		2	1			2
Other terrestrial fats?, Ruminant carcass fats?						3

### Norwegian coastal areas

**Figure 3:**
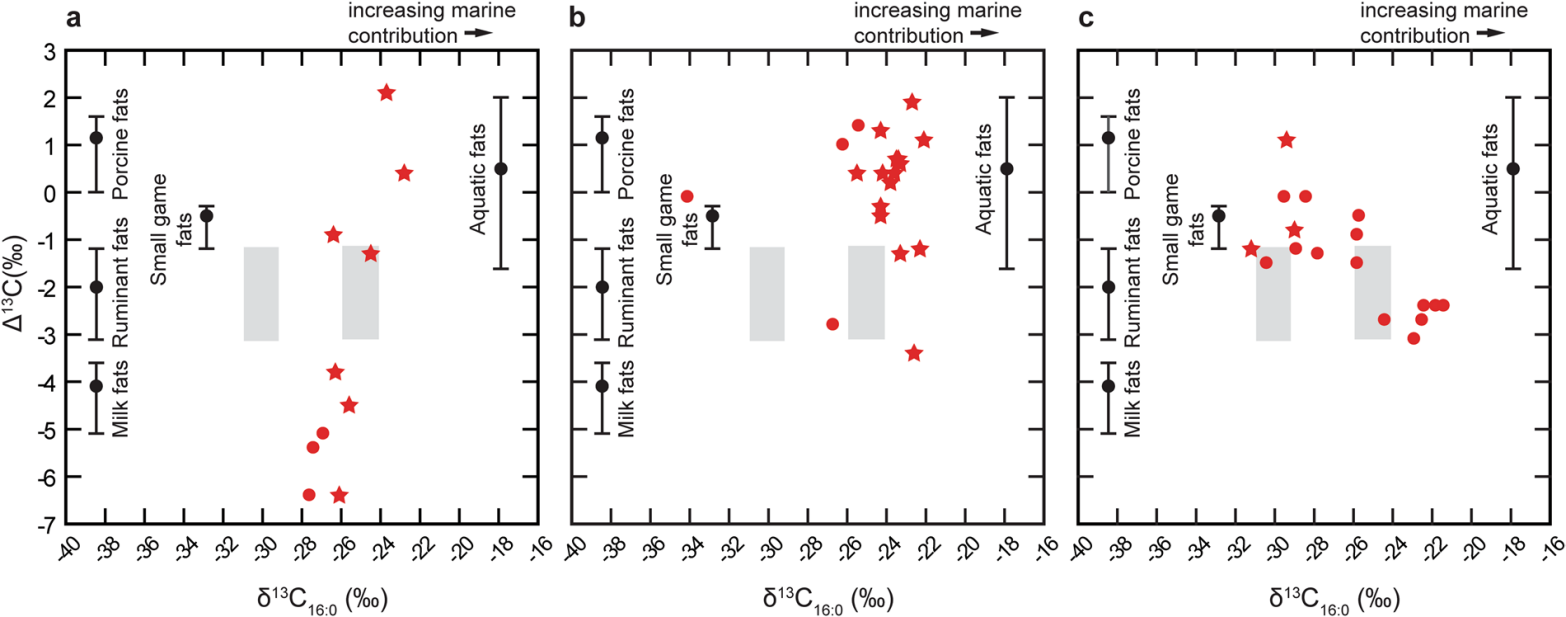
Panels showing the Δ^13^C (δ^13^C_18:0_-δ^13^C_16:0_) proxy for the different studied economic groups. (**a**) Coastal agrarian groups using dairy and marine products, (**b**) coastal hunter-gatherers, with a diet based on marine mammals and fish, and (**c**) interior hunter-gatherers with a diverse food procurement pattern consisting of terrestrial animals and fresh water fish. The organic residues from vessel plotting to the RHS contained a greater marine component compared to those plotting more to the LHS. The stars represent lipid residues that contained biomarkers of marine/aquatic origin, and the circles represent the fats that originated from terrestrial resources. The grey rectangles indicate the range of δ^13^C_16:0_ values of modern Eurasian elk fats (on the left) and reindeer/wild forest reindeer fats (on the right)^49^.

**Figure 4:**
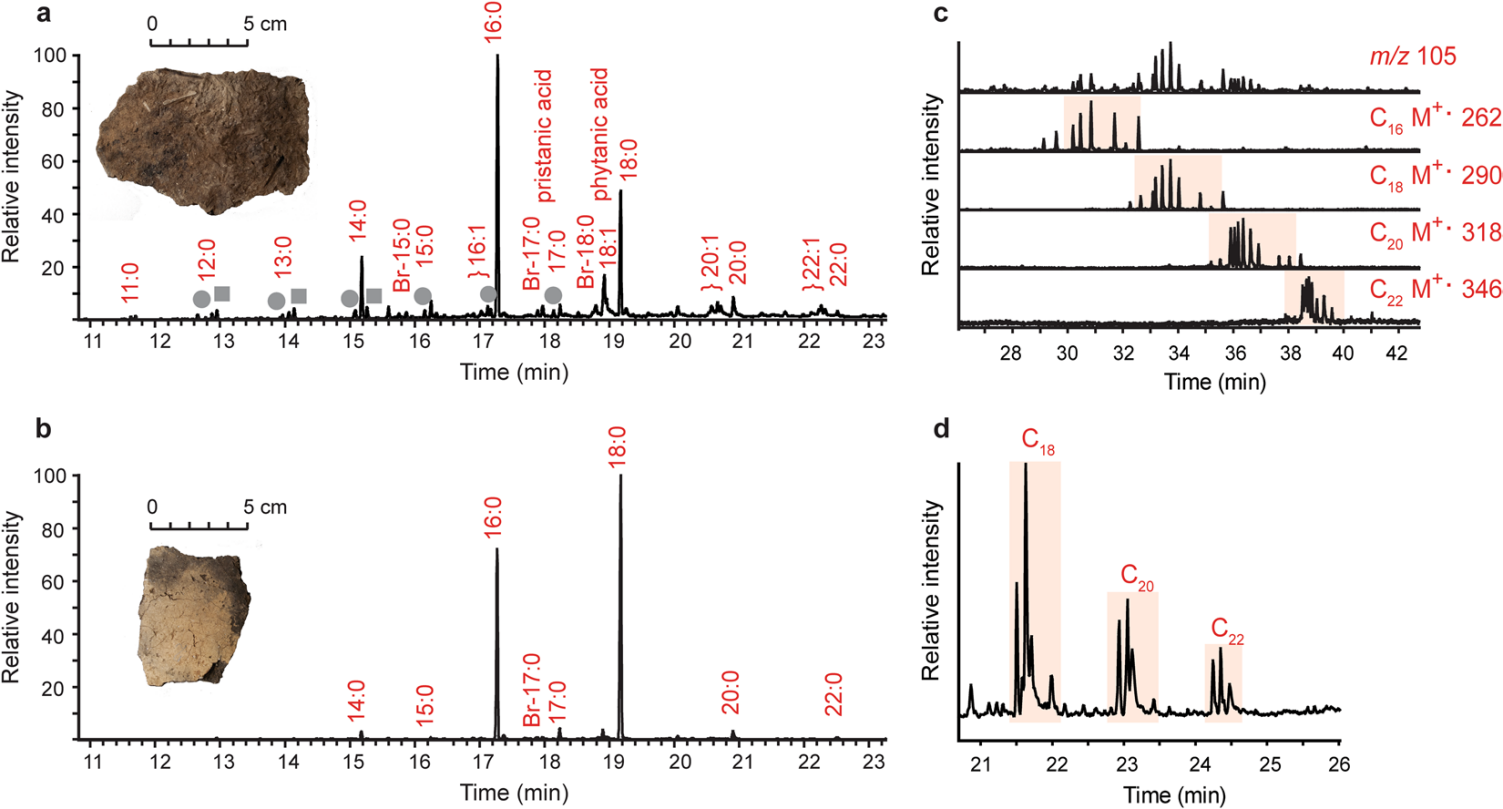
Partial gas chromatogram of fat residues extracted from Kjelmøy potsherds. (**a**) Sherd SÄR10B from a coastal site and (**b**) SÄR14 from interior Norway. Grey circles indicate alkanes, squares indicate α,ω-dicarboxylic acids, and Br indicates branched chain fatty acid. (**c**) A partial selected ion monitoring (SIM) chromatogram of SÄR10B shows the base peak *m/z* 105 of the APAAs and the distribution of APAAs with chain lengths C_16_–C_22_, and (d) the partial SIM chromatogram of SÄR10B shows the distribution of DHYAs. C_18_, C_20_, and C_22_ indicate the chain lengths of the DHYAs. The findings of APAAs and DHYAs strongly indicate the processing of marine commodities in vessel SÄR10B.

Of the nine Risvik sites investigated, sherds from five sites yielded organic residues that were likely to derive from dairy fats. Of these, lipid residues from three vessels, based on their Δ^13^C (δ^13^C_18:0_-δ^13^C_16:0_) proxy, are dominated by dairy fats. Ancient fat residues from three other vessels exhibited APAAs, DHYAs, and in one case, phytanic acid (Supplementary Table S1). Nonetheless, the Δ^13^C values of these residues indicate a predominantly dairy fat component (Fig. 3); thus, these residues are likely to have resulted from the use of these vessels to process both marine and dairy products. In four sherds, the δ^13^C values, detection of APAAs, DHYAs (Fig. 4) and isoprenoid fatty acids indicate the vessels were used to process predominantly marine products. The high frequency of marine product biomarkers in the pottery fits the interpretation^19^ that animal husbandry played a minor role in fulfilling the nutritional demands of the people using Risvik pottery, with their economy otherwise based mostly on hunting and gathering.

Kjelmøy, IT, and mica and shell tempered pottery sherds from these northern coastal hunter-gatherer sites yielded residues that contained C_16_–C_22_ APAAs, long-chain DHYAs, and IFAs (Supplementary Table S1), thereby indicating a substantial marine contribution to the typical diet. Nevertheless, these people did not solely exploit the marine environment for food, with seven of the lipid residues shown as originating from ruminant animals and/or possibly other terrestrial animals (Table 1).

### Interior Norway and Finland

None of the studied vessels from the inland sites contained DHYAs with chain-lengths of C_20_–C_22_; even though APAAs and IFAs were detectable in the residues. The carbon isotope values and specific biomarker compositions of three sherds suggest the processing of both terrestrial and aquatic products (Supplementary Table S1). The results obtained from the organic residue analyses of the other vessels from the inland sites were very consistent. Ruminant animal fats, most likely originating from reindeer, dominated the pottery from Virdejávri 106, while the Sär 2 vessels contained lipid residues of other terrestrial animals and fish, together with ruminant fat residues (Table 1, Supplementary Table S1).

## Discussion

One of our goals was to interpret animal subsistence based on the lipid residues found in the pottery in tandem with zooarchaeological evidence. However, in some areas of Fennoscandia, especially at Finnish prehistoric sites, the preservation of animal bone is often very poor with only small burnt fragments recovered. Further, due to the high fragmentation rates and the low number of identified bone fragments per site, the assemblages are often relatively uninformative, although seen as broadly reflecting regional economies^20–22^. Assemblages were comprised of burnt bone fragments from wild mammals, birds, and fish. The species represented were typical of those expected for fauna of Boreal zones inland hunter-gatherer communities^20,22^.

Bone preservation at coastal sites in North Norway is generally better than in Finland, and many sites contain large unburned assemblages^23,24^. Identified species from the coastal Early Metal Period bone assemblages include fish and marine mammals; however, terrestrial species are also widely represented. The general assessment indicates that hunting and fishing predominated; however, fragments of teeth and bones of cattle (*Bos taurus*) and sheep or goats (*Ovis aries*/*Capra hircus*) have been recovered from the Risvik pottery settlements and other Early Metal Period coastal settlements in North Norway^25–27^. In addition, well-dated traces of cereal cultivation (pollen and grains) have also been identified at coastal sites from this period^28^. Zooarchaeological assemblages from the coastal early agrarian sites (Table 2), are in agreement with the results obtained from organic residue analysis.

**Table 2:** Identified bone fragments from the sites included in this study from Norway (Number of Identified Specimens, NISP): Sandvika^37^, Kirkhelleren^38^, Mestersanden^24^, Mortensnes shows bone material only from those parts of the sites that date back to the Late Stone Age/Early Metal Period^39^. Number with # = NISP was greater than that given here, but exact numbers are not given. No zooarchaeological data were available from the other studied Norwegian sites. More detailed information is presented in Supplementary Table S2.

	**Risvik Pottery**		**Kjelmøy Pottery**	
	**Tromsø Sandvika**	**Træna Kirkhellaren**	**Sørvaranger Mestersanden**	**Mortenses Nesseby**
Domesticated animals	2	present	0	0
Wild ruminant animals	0	present	61#	16
Wild non-ruminant animals	0	present	44	3
Marine mammals	1	present	238#	140
Birds	0	present	872	8
Fish	present	present	524	16

The dairy fat residues and bones of domesticated ruminants recovered from the Kirkhellaren site are particularly interesting, since the site is located on a remote island more than 40 km from the coast. This discovery of dairy fats provides clear evidence of agrarian groups having populated even the most challenging environments. Surprisingly, no indication of terrestrial carcass fats was observed in the lipid residues recovered from the pottery of the coastal early agrarian settlements, even though terrestrial animal remains were present in the zooarchaeological assemblages. The lack of terrestrial carcass fats could result from sampling bias or indicate specialised food preparation practises, wherein terrestrial carcass products were not prepared in ceramic vessels, instead were smoked, dried, or processed in containers other than ceramic vessels. The major conclusion, however, is that the early farmers of the coastal zone used ceramic vessels mainly for processing marine and dairy products.

Although reindeer (*Rangifer tarandus*) herding is a characteristic of the economy of the North Fennoscandian people today, it is a relatively recently introduced phenomenon, dating back only to the historical period^3,29^. In prehistoric times, small numbers of tame reindeer could have been used as decoy animals for reindeer hunting and perhaps even for transportation and milking^30,31^. Thus, residues of dairy fats detected in the pottery could hypothetically have derived from the reindeer, rather than from cattle, sheep, or goats. However, the fact that dairy fats were detected in the Risvik rather than in the Kjelmøy pottery makes this interpretation unlikely, since reindeer were far more important to the people using the latter type of pottery.

In North Norway coastal hunter-gatherer groups lived north of the early agrarian societies. The bone assemblages from their sites comprise a wide range of species, mostly marine mammals and, fish, including seals (*Phocidae*) whales (*Cetacea*), saithe (*Pollachius virens*), and cod (*Gadus morhua*). However, terrestrial species are also represented (Table 2), for example reindeer and European beaver (*Castor fiber*). A total of 28 different species of birds have also been identified (Supplementary Table S2). The presence of terrestrial fats in coastal hunter-gatherer sherds indicates a versatile use of resources largely in-line with the zooarchaeological findings and indicate that the coastal hunter-gatherers based their diet on both marine and terrestrial resources.

The zooarchaeological assemblages from the interior hunter-gatherer sites comprised also terrestrial animals (Table 3), including: European beaver, Eurasian elk (*Alces alces*), and reindeer. Fish are also present, including pike (*Esox lucius*) and those of the carp family (Cyprinidae, Supplementary Table S3). Bones of the European beaver were recovered in large quantities from these sites, suggesting they were hunted for food and not just for castoreum or for fur. Based on the dominance of terrestrial fauna in the zooarchaeological assemblages, it is presumed that the pottery from the interior of Fennoscandia would have been used to process terrestrial animal products, rather than products derived from freshwater aquatic organisms.

**Table 3:** Identified bone fragments from the sites included in this study from Finnish SÄR2 sites (Number of identified specimens NISP): Kalmosärkkä (KM 14504, KM 14289, KM 14830^40,41^), Kellolaisten Tuli (KM 14246, 14505, 14831^42^, Neitilä 4 (KM 15671, 16145, 16553^43–45^). More detailed information is presented in Supplementary Table S3.

	**Suomussalmi Kalmosärkkä S**	**Suomussalmi Kalmosärkkä N**	**Suomussalmi Kellolaisten Tuli**	**Kemijärvi Neitilä 4**
Domesticated animals	0	0	0	0
Wild ruminant animals	2	14	47	26
Wild non-ruminant animals	8	59	66	189
Marine mammals	0	0	0	0
Birds	0	3	3	1
Fish	21	119	72	84

The composition of the zooarchaeological assemblages and the results of the organic residue analyses of the pottery from interior sites were in accordance with each other with the fat residues indicating cooking/storing of ruminant products, together with that of other terrestrial animals and minor contributions from freshwater resources. The extensive use of bifacial arrow points in the interior during the Early Metal period is considered a potential indicator of intensified reindeer hunting^32^. This result agrees with the finding of ruminant carcass fat residues in vessels from sites located inland, such as Virdejávri 106 (Supplementary Table S1).

The Norwegian coast is ice-free during winter and is generally considered to be a more productive area for both marine and terrestrial resources than the North Fennoscandian interior^33^. The compositional differences in the lipid residues in pottery between the coastal and the interior hunter-gatherer sites reflects the different environmental productivities of the regions. The more challenging environment of the interior probably required a more differentiated subsistence economy, i.e., groups in the interior had to use most of available resources. In the more hospitable and economically productive environment of the Norwegian coastal zone, food procurement strategies clearly became more specialised. Short-term or seasonal sites in the interior appear to display less diversity in their food-ways compared to the more permanent settlement sites^34^. This is seen, for example, in the dominance of ruminant fats in the pottery from Virdnejávri 106, where lack of diversity in fat residues is likely due to the seasonal occupation of the site rather than a specialised diet of the people.

Based on the findings of domesticated animal bones and cereals, and our results from the analysis of lipid residues in pottery, the coastal groups using Risvik pottery were clearly gradually transitioning towards farming. Among these groups, hunting, fishing and farming were practised simultaneously. In contrast, for the communities living further north in the coastal zone and in the interior, agriculture and animal husbandry remained for a long time an available, yet unrealized, option. Living in environments with less favourable farming conditions these groups chose to continue the economically less-risky hunting and gathering.

Overall, these new results show that the North Fennoscandian economic landscape of the last millennium BC can be divided into three new and distinct categories: (i) coastal agrarian communities who based their food economy on dairy and marine products; (ii) coastal hunter-gatherers with an emphasis on marine mammals and fish; and (iii) interior hunter-gatherers with more a diverse food procurement pattern that consisted of terrestrial animals and freshwater fish.

With regard to explaining the transition to farming among coastal groups in North Norway, it seems evident that economic factors alone do not suffice. Especially since marine resources were abundant and all coastal groups could have based their economies on fishing and marine mammal hunting. Nevertheless, in some areas potential drivers toward animal husbandry may have included economic and nutritional gains, thereby encouraging hunter-gatherer groups to move toward a dairy-oriented economy. It has also been suggested that animal domestication could have been introduced simply to increase the quantity of meat in the diet^35^. Based on the analysed lipid residues found, this seems unlikely, as only aquatic and dairy fats were observed in the studied coastal early agrarian potsherds. However, as stated earlier, the ruminant carcass elements could have been processed in different types of vessels than pottery.

Fishing continued to be important to coastal farming sites in North Norway up to modern times. Due to the richness of natural coastal resources, any possible failure in cultivation and animal husbandry would not have led to a nutritional crisis. Based on this understanding, the rich natural resources of coastal North Norway may be seen as enabling some communities to experiment with and adopt the economically riskier food procurement strategies of cereal cultivation and animal husbandry. This dynamic in food procurement practises can also be seen as related to the ongoing processes of cultural differentiation, wherein the adoption of farming also implied a cultural and social orientation towards South Scandinavian farming societies – and simultaneously a wish to distinguish themselves from the former foraging peers in the north. In other words, the groups that were using Risvik pottery could have adopted farming or at least dairying also as a mean to strengthen their economic and social relationships with other agrarian groups^19^.

Thus, the potential cultural gains of farming should not be underestimated, especially as a means of expressing cultural affiliation and difference. The increased interaction with the southern and more hierarchical farming communities may have made this kind of “mimicking” advantageous and perhaps even imperative in order to enhance the contacts. Thus, animal husbandry would have been a common mediator occurring between Arctic early agrarian groups and South Scandinavian Nordic Bronze Age communities^19^. Neither should one forget the possible significance of farming products for luxury consumption in social and ritual gatherings.

In summary, based on the lipid biomarkers preserved in ancient lipid residues in ceramic food processing vessels, coastal and interior hunter-gatherer groups in North Fennoscandia chose divergent food procurement strategies due to both differing environmental factors and contrasting socio-cultural needs. The new organic residue evidence thus provides novel insights into the divergent food habits of prehistoric societies in the region. We have shown for the first-time that dairying was practiced in Arctic Norway by the early agrarian groups. However, animal domestication was only part of the economic base, as marine resources continued to play a critical role in the food procurement cycle. Even though some of the coastal groups had already included dairy fats into their diet, the food procurement patterns were quite similar in the coastal area. Thus, the richness of natural resources also may actually have delayed the transition to a fully agrarian community in northern areas.

## Methods

### Lipid analysis

Sub-samples of surface-cleaned sherds (1–2 g) were extracted using direct methanolic acid extraction^36^. After the extraction, an aliquot was treated with *N*,*O*-bis(trimethylsilyl)trifluoroacetamide (20 µl) in readiness for high-temperature GC (HTGC) and GC-MS and GC-C-IRMS analyses.

### High temperature gas chromatography (HTGC)

HTGC analyses were performed using Agilent Technologies 7890 A GC. Diluted samples were introduced via on-column injection. The column was DB-1ht (15 m × 0.32 mm i.d., coated with dimethylpolysiloxane, film thickness, 0.10 μm, Agilent Technologies). The oven temperature was held isothermally for 2 min at 50 °C and then increased to 350 °C at 10 °C/min, followed by an isothermal hold at 350 °C for 10 min. The flame ionization detector (FID) was set to a temperature of 350 °C. Helium was used as a carrier gas and maintained at constant flow of 4.6 ml/min. The peaks were identified by their retention times, and quantification was achieved by referencing the internal standard method.

### Gas chromatography-mass spectrometry (GC/MS)

The GC/MS analyses of fatty acid methyl esters (FAMEs) were performed using a Finnigan Trace MS quadrupole mass spectrometer, coupled to a Trace GC. Diluted samples were introduced using a PTV injector in splitless mode onto a HP-1 (50 m × 0.32 mm i.d. fused-silica capillary column coated with dimethylpolysiloxane, film thickness, 0.17 μm, Agilent Technologies). The GC oven temperature was programmed as follows: 50 °C for 2 min, then to 300 °C at 10 °C/min, and a hold at 300 °C for 10 min. Helium was used as the carrier gas and maintained at constant flow 5 ml/min. The MS was operated in electron ionization (EI) mode (70 eV) with a GC/MS interface temperature of 250 °C and a source temperature of 200 °C. The emission current was 50 μA, and the MS was set to acquire in the range of *m/z* 50–650 at two scans per second. Peaks were identified on the basis of their mass spectra using Thermo Xcalibur 3.0.63 software.

The structural identification of APAAs was carried out with polar GC/MS (VF-23ms, 60 m × 0.32 mm i.d. fused-silica capillary column coated with cyanopropyl film thickness, 0.15 μm, Agilent Technologies). The initial oven temperature was 50 °C with an evaporation phase of 1 min, followed by a transfer phase from 50 °C to 250 °C at 10 °C/min, then by an isothermal hold at 250 °C for 10 min. The MS was operated in EI mode (70 eV) with a GC/MS transfer line temperature of 250 °C and a source temperature of 200 °C. Specific ions for APAAs were detected using selected ion monitoring (GC/MS-SIM, scanning for the ions *m/z* 105, 262, 290, 318, and 346). Identification of DHYAs was carried out by GC/MS (HP-1, 50 m × 0.32 mm i.d. fused-silica capillary column, coated with dimethylpolysiloxane, film thickness, 0.17 μm, Agilent technologies and RTX-1, 50 m × 0.32 mm i.d. fused-silica capillary column coated with dimethylpolysiloxane, film thickness, 0.17 μm, Restek). The MS was operated in EI mode (70 eV) with a GC/MS transfer line temperature of 300 °C and a source temperature of 300 °C. The initial port temperature was 50 °C for 1 min followed by a temperature programme from 50 °C to 300 °C at 10 °C/min, followed by an isothermal hold at 300 °C for 10 min. Specific ions for DHYAs were detected using SIM mode (scanning for ions *m/z* 159, 187, 215, 243, 259, 287, 315, 443, 471, 499).

### Gas-chromatography-combustion-isotope ratio mass spectrometry (GC-C-IRMS)

The carbon isotope compositions of the C_16:0_ and C_18:0_ fatty acids were determined by using gas chromatography-combustion-isotope ratio mass spectrometry (GC-C-IRMS) and an Agilent Technologies 7890 A GC coupled to an IsoPrime 100 (70 eV, three faraday cup collectors *m/z* 44, 45, and 46) via an IsoPrime GC5 combustion interface with a CuO and silver wool reactor maintained at 850 °C. Diluted samples were introduced using a PTV injector in the splitless mode onto a HP-1 (50 m × 0.32 mm i.d. fused-silica capillary column coated with dimethylpolysiloxane, film thickness, 0.17 μm, Agilent Technologies). The GC oven temperature was programmed from 40 °C, following an isothermal hold for 1 min, to 300 °C at 10 °C/min, followed by an isothermal hold at 300 °C for 10 min. Each sample was run in duplicate.

### Data availability

The data that support the findings of this study are available from the corresponding author upon request.

## Electronic supplementary material

Supplementary data

Supplementary Table S1
