# Supplementary material for: Contrasting patterns of prehistoric human diet and subsistence in northernmost Europe
Source: Sci Rep. 2018 Jan 18;8:1148. doi: 10.1038/s41598-018-19409-8 (PMC5773502; doi:10.1038/s41598-018-19409-8)
Supplement: Supplementary file 1 — Supplementary data [file 41598_2018_19409_MOESM1_ESM.pdf]

Contrasting patterns of prehistoric human diet and subsistence in northernmost Europe

Mirva Pääkkönen, Auli Bläuer, Bjørnar Olsen, Richard P. Evershed, Henrik Asplund

Supplementary information

Supplementary Table S1. Description of the studied sherds. The following abbreviations were used to describe the biomarker composition of the vessels: FFA – free fatty acids, APAAs – ω-(*o*-alkylphenyl)alkanoic acids; TMTD – 4,8,12-trimethyltridecanoic acid; Phy – phytanic acid; Pris – pristanic acid, DHYA – dihydroxy acids; ? – uncertain identification of biomarker due to low concentration. The abbreviations A and O describe the type of temper material, A – asbestos and O – other temper. * = sherd resembling Risvik pottery. SÄR16 is represented as a strike through since the isotopic value is likely to be contaminated, and the vessel was not included in the discussion. Vessels without interpretation were screened with HTGC, but they either contained no fatty acids or they contained phthalate esters originating from plasticiser constituting a modern contamination introduced during storage. Thus, the vessels were not studied further with GC/MS and GC-C-IRMS. The radiocarbon calibration used the OxCal v3.10 program[1, 2] and the IntCal13 calibration data[3].

Supplementary Table S2. Identified bone fragments from the sites included in this study from Norway (Number of Identified Specimens, NISP): Sandvika[4], Kirkhelleren[5], Mestersanden[6], Mortensnes shows bone material only from those parts of the sites that date back to the Late Stone Age/Early Metal Period[7]. * = NISP not given. Number with # = NISP was greater than that given here, but exact numbers are not given. x = NISP not available. No zooarchaeological data were available from the other studied Norwegian sites.

	**Risvik Pottery**	**Risvik Pottery**	**Kjelmøy Pottery**	**Kjelmøy Pottery**
**Species/Site**	**Tromsø Sandvika**	**Træna Kirkhellaren**	**Sørvaranger Mestersanden**	**Mortenses Nesseby**
Cattle (*Bos taurus*)		**x**		
Sheep/goat (*Ovis aries/Capra hircus*)	2	x		
Reindeer (*Rangifer tarandus*)			61#	16
Eurasian elk (*Alces alces*)		x		
Red deer (*Cervus elaphus*)		x		
Artic hare (*Lepus timidus*)				1
European beaver (*Castor fiber*)			33	
Pine marten (*Martes martes*)			3	
Red fox (*Vulpes vulpes*)		x	1	
Otter (*Lutra lutra*)		x	1	1
Rat (Rattus sp.)			6	
Rodentia				1
Harp seal (*Pagophilus groenlandicus*)			61#	37
Harbor seal (*Phoca vitulina*)		x	16#	4
Grey seal (*Halichoerus grypus*)		x	7	
Grey seal/Harp seal (*Halichoerus grypus/Phoca groenlandicus*)		x		
Grey seal/Harbour seal (*Halichoerus grypus/Phoca vitulina*)		x		
Seal (Phocidae)	1	x	138	78
Walrus (*Odobenus rosmarus*)			1	1
Harbour porpoise (*Phocoena phocoena*)			11	4
Whale (Cetacea)			4	16
European shag (*Phalacrocorax aristotelis*)				2
Black guillemot (*Cepphus grylle*)		x	1	
Common murre (*Uria aalge*)		x	40	
Atlantic puffin (*Fratercula arctica*)		x	123	

Razorbill (*Alca torda*)		x	10	
Great auk (*Penguinus impennis*)		x	3	
Little auk (*Alle alle*)		x		
Northern fulmar (*Fulmarus glacialis*)		x	41	
Common eider (*Somateria mollissima*)		x	13	
King eider (*Somateria spectabilis*)		x	3	
Common merganser (*Mergus merganser)*			7	
Common goldeneye (*Bucephala clangula*)			4	
Velvet Scoter (*Melanitta fusca*)		x		
Long -tailed duck (*Clangula nyemalis*)			1	
Whooper swan (*Cygnus cygnus*)		x	4	
Greylag goose (*Anser anser*)		x	4	
Yellow-billed loon (*Gavia adamsii*)		x		
Loon (Garia sp.)			6	
Northern gannet (*Sula bassana*)		x		
Dunlin (*Calidris alpina*)		x		
Great Skua (*Stercorarius skua*)		x		
European herring gull (*Larus argentatus*)		x	12	
Great black-backed gull (*Larus marinus*)		x	18	
European herring gull/ Greal black-backed gull (*Larus argentatus/Larus marinus*)		x		
Lesser black-backed gull (*Larus fuscus*)		x		
Mew gull (*Larus canus*)		x	2	
Black-legged kittiwake (*Rissa tridactyla*)		x	1	
Mew gull/Black-legged kittiwake (*Larus canus/Rissa tridactyla*)		x		
Arctic tern (*Sterna paradisaea)*		x	1	
Eurasian curlew (*Numenius arquata*)		x		
Eurasian oystercatcher *(Haematopus ostralegus)*		x	1	
Great cormorant (*Phalacrocorax carbo*)		x	94	1
European shag (*Phalacrocorax aristotelis*)		x	23	
White-tailed eagle (*Haliaeetus albicilla*)		x	15	

Golden eagle (*Aquila chrysaetos*)		x		
Common raven (*Corvus corax*)		x	2	5
Carrion crow (*Corvus corone*)		x		
Rough-legged buzzard (*Buteo lagopus*)			4	
Great grey owl (*Strix nebulosa*)			2	
Western Capercaillie (*Tetrao urogallus*)			1	
Rock ptarmigan (*Lagopus mutens*)		x		
Willow grouse (*Lagopus lagopus*)		x		
Rock ptarmigan/Willow grouse (*Lagopus lagopus/Lagopus mutens*)		x		
Bird	2		436	
Saithe (*Pollachius virens*)		x	407	7
Cod (*Gadus morhua*)		x	81	9
Saithe/Cod (*Pollachius virens/Gadus morhua*)		x		
Haddock (*Melanogrammus aeglefinnus*)			16	
Common ling (*Molva molva*)		x	3	
Cusk (*Brosme brosme*)		x	7	
Commong ling/Cusk (*Brosme brosme/Molva molva*)		x		
Atlantic halibut (*Hippoglossus hippoglossus*)		x	10	
Atlantic wolffish (*Anarhichas lupus*)		x		
Fish	present*			

24

Supplementary Table S3. Identified bone fragments from the sites included in this study from Finland (Number of identified specimens NISP): Kalmosärkkä (KM 14504, KM 14289, KM 14830[8, 9]), Kellolaisten Tuli (KM 14246, 14505, 14831[10], Neitilä 4 (KM 15671, 16145, 16553[11–13]).

**Species/Site**	**Suomussalmi Kalmosärkkä S**	**Suomussalmi Kalmosärkkä N**	**Suomussalmi Kellolaisten Tuli**	**Kemijärvi Neitilä 4**
European beaver (*Castor fiber*)	7	55	62	170
Beaver?	1			17
European elk (*Alces alces*)		1	5	18
Elk?		7	14	4
Reindeer (*Rangifer tarandus*)		1	1	1
Reindeer?			10	
Elk/Reindeer	1	5	17	3
Elk/reindeer?	1			
Bear (*Ursus arctos*)			1	2
Pine marten? (*Martes martes?*)		3	2	
Dog/wolf? (Canis sp.?)		1		
Mustelidae?			1	
Bird		1	3	
Bird?				
Divers? *(Gavia?*)				1
Grouse family		2		
Pike (*Esox lucius*)	6	50	27	20
Pike?	1		2	
Perch *(Perca fluviatilis*)				2
Perch?		1		
Carp family (Cyprinidae)	6	29	9	
Salmon family? (Salmonidae?)		1		
Fish	8	38	34	62

References

1. Bronk Ramsey, C. Radiocarbon calibration and analysis of stratigraphy: the OxCal program. *Radiocarbon* **37,** 425-430 (1995).

2. Bronk Ramsey, C. Development of the radiocarbon calibration program. *Radiocarbon* **43,** 355–364 (2001).

3. Reimer, P. J. *et al*. IntCal13 and Marine13 radiocarbon age calibration curves 0–50,000 years cal BP. *Radiocarbon* **55,** 1869–1887 (2013).

4. Denham, S. D. *Animal Bone Fragments from Sandvika, Tromsø Commune* (University of Bergen, 2014).

5. The University Museum of Bergen's osteological collections. https://reg.app.uib.no/apex/f?p=608:1:2415677446625 (2017).

6. Olsen, B. *Stabilitet og Endring. Produksjon og Samfunn i Varanger 800 f.Kr.–1700 e.Kr.* (University of Tromsø, 1984).

7. Schanche, K. *Mortensnes en Boplass i Varanger. Et Studie av Samfunn og Materiell Kultur Gjennom 10.000 År* (University of Tromsø, 1988).

8. Fortelius, M. *Suomussalmi Kalmosärkkä 14244, 14504, 14829 & 14830. Osteologinen Analyysi* (Finnish National Board of Antiquities, 1980).

9. Mannermaa, K. Birds in Finnish prehistory. *Fennoscandia Archaeologica* **XX,** 3–39 (2003).

10. Fortelius, M. *Suomussalmi Kellolaisten Tuli 14246, 14505 & 14831. Osteologinen Analyysi* (Finnish National Board of Antiquities, 1980).

11. Ukkonen, P. *Kemijärvi Neitilä 4 (KM 15671)/Sarvas 1962. Luuanalyysi 22.7.1996* (Finnish National Board of Antiquities, 1996).

12. Ukkonen, P. *Kemijärvi Neitilä 4 (KM 16145)/Sarvas 1963. Luuanalyysi 22.7.1996* (Finnish National Board of Antiquities, 1996).

13. Ukkonen, P. *Kemijärvi Neitilä 4 (KM 16553)/Sarvas 1964. Luuanalyysi 22. 7.1996* (Finnish National Board of Antiquities, 1996).
